# Maternal immunization in Malawi: A mixed methods study of community perceptions, programmatic considerations, and recommendations for future planning

**DOI:** 10.1016/j.vaccine.2019.06.020

**Published:** 2019-07-26

**Authors:** Jessica A. Fleming, Alister Munthali, Bagrey Ngwira, John Kadzandira, Monica Jamili-Phiri, Justin R. Ortiz, Philipp Lambach, Joachim Hombach, Kathleen M. Neuzil, Maria Stepanchak, Niranjan Bhat

**Affiliations:** a Center for Vaccine Innovation and Access, PATH, 2201 Westlake Ave, Suite 200, Seattle, WA 98121, USA; b The Centre for Social Research, University of Malawi, PO Box 280, Zomba, Malawi; c Malawi Polytechnic, Private Bag 303, Chichiri, Blantyre 3, Malawi; d Initiative for Vaccine Research, World Health Organization, Appia 20, 1211, Geneva 27, Switzerland; e Global Alliance to Prevent Prematurity and Stillbirth, 19009 33rd Ave W #200, Lynnwood, WA 98036, USA

**Keywords:** Maternal immunization, Malawi, Maternal tetanus toxoid and maternal influenza vaccine, Operational research

## Abstract

**Background:**

Safe, effective vaccines are given to pregnant women to protect their infants and/or themselves against certain infectious agents; however, apart from tetanus vaccination, maternal immunization in low- and middle-income countries (LMICs) remains low. Tetanus toxoid vaccine is integrated into antenatal care services in Malawi with high coverage and provides an opportunity to identify factors that facilitate successful immunization delivery to pregnant women in LMICs.

**Methods:**

PATH and the University of Malawi’s Centre for Social Research conducted a mixed-methods study in 2015 to document community perceptions of maternal immunization, using tetanus vaccine as an example, and to identify factors perceived to be important to successfully introducing other maternal vaccines, such as influenza vaccine, in Malawi. We conducted 18 focus group discussions with pregnant and recently pregnant women and their family members and 76 semi-structured interviews with pregnant and recently pregnant women, community leaders, health workers, public health program managers, non-governmental partners, and policy makers.

**Results:**

We identified factors perceived to support the introduction of new maternal vaccines, including strong maternal vaccine acceptance in the community, an existing strategy for maternal tetanus vaccine delivery, and positive health workers’ views about the introduction of additional maternal vaccines. Potential challenges to adoption and acceptance included identifying and tracking the target population and monitoring adverse events, and the need to ensure operational capacity of the health system to support the introduction and wide-scale use of an additional vaccine. For influenza vaccine specifically, additional challenges included limited awareness of influenza disease and its low prioritization among health needs.

**Conclusions:**

Lessons from the successful delivery of maternal tetanus immunization in Malawi may be informative for similar countries considering new vaccines for pregnant women or striving to optimize the delivery of those currently provided.

## Highlights

• Health workers and community members expressed high acceptance of maternal vaccines.
• Malawi’s strong tetanus vaccine delivery system may benefit new maternal vaccines.
• Community members and policy makers have low awareness of influenza disease burden.

## 1 Introduction

Vaccines against tetanus, pertussis, and influenza are recommended in certain contexts for pregnant women by the World Health Organization (WHO), and new vaccines for use during pregnancy are under development [1], [2], [3], [4], [5]. In low- and middle-income countries (LMICs), influenza and pertussis vaccines are seldom used [1], [6]; in contrast, tetanus toxoid-containing vaccines (TTV) have been adopted for pregnant women as part of comprehensive programs in countries striving to eliminate maternal and neonatal tetanus [7]. WHO has emphasized that national immunization policies be determined based on country priorities and data review, and WHO maternal immunization policy recommendations are not universal, but rather depend on the particular national context [1], [2], [3]. Reliance on national or regional data may be necessary for countries to determine potential program impact and feasibility. Systematic reviews of research relevant to operationalizing maternal immunization have identified substantial data gaps in LMICs [8], [9], [10], [11]. In 2016, the WHO Strategic Advisory Group of Experts (SAGE) on Immunization called for more research to “generate generalizable data on the best ways to integrate maternal immunization into routine antenatal care in low resource settings” [12]. Lessons can be learned from LMICs that have successful maternal tetanus vaccine programs and that have eliminated maternal and neonatal tetanus.

PATH, an international, non-governmental organization, partnered with WHO to conduct two studies to identify opportunities and challenges to the introduction and delivery of maternal immunization in LMICs. We sought to conduct studies in one middle-income country in Latin America with high maternal influenza immunization coverage and one lower-income country in sub-Saharan Africa with high maternal tetanus immunization coverage. We sought advice from WHO, WHO Regional Offices, and immunization experts to identify countries that would be amenable to our research and whose experiences would be informative for other countries in their sub-regions and regions. The results from our study in El Salvador have been published [13]. We chose Malawi as the site for our second study, which we conducted with the University of Malawi’s Centre for Social Research.

When this study was conducted in 2015, Malawi provided TTV to pregnant women through routine antenatal care (ANC) services; tetanus-diphtheria vaccine replaced TTV in 2017. Tetanus vaccine is provided by nurses in public and private health facilities and by lay community health workers through community outreach, commonly located in churches, schools, and market places. The 2015-16 Malawi demographic health survey reports 73% of women received two or more doses of TTV in their last pregnancy and 90% of women’s most recent births were protected against neonatal tetanus by routine childhood immunization and immunization during pregnancy [14]. The experience of maternal tetanus immunization in Malawi may inform successful introduction of additional maternal vaccines, such as influenza vaccine, and inform improved delivery of maternal immunization in general in Malawi and other similar countries.

The objectives of this study were to determine community perceptions of disease transmission and prevention, vulnerability, and health priorities of pregnant women in Malawi; to understand factors associated with acceptance of maternal immunization, health care decision-making for pregnant women, and facilitators and challenges to receiving ANC, including vaccination; and to identify programmatic and operational factors perceived as important to the introduction and uptake of new maternal vaccines, specifically seasonal influenza vaccine, which is currently not routinely provided in Malawi, in an effort to inform future country-level maternal immunization implementation.

## 2 Methods

### 2.1 Study sites and characteristics

**Fig. 1:**
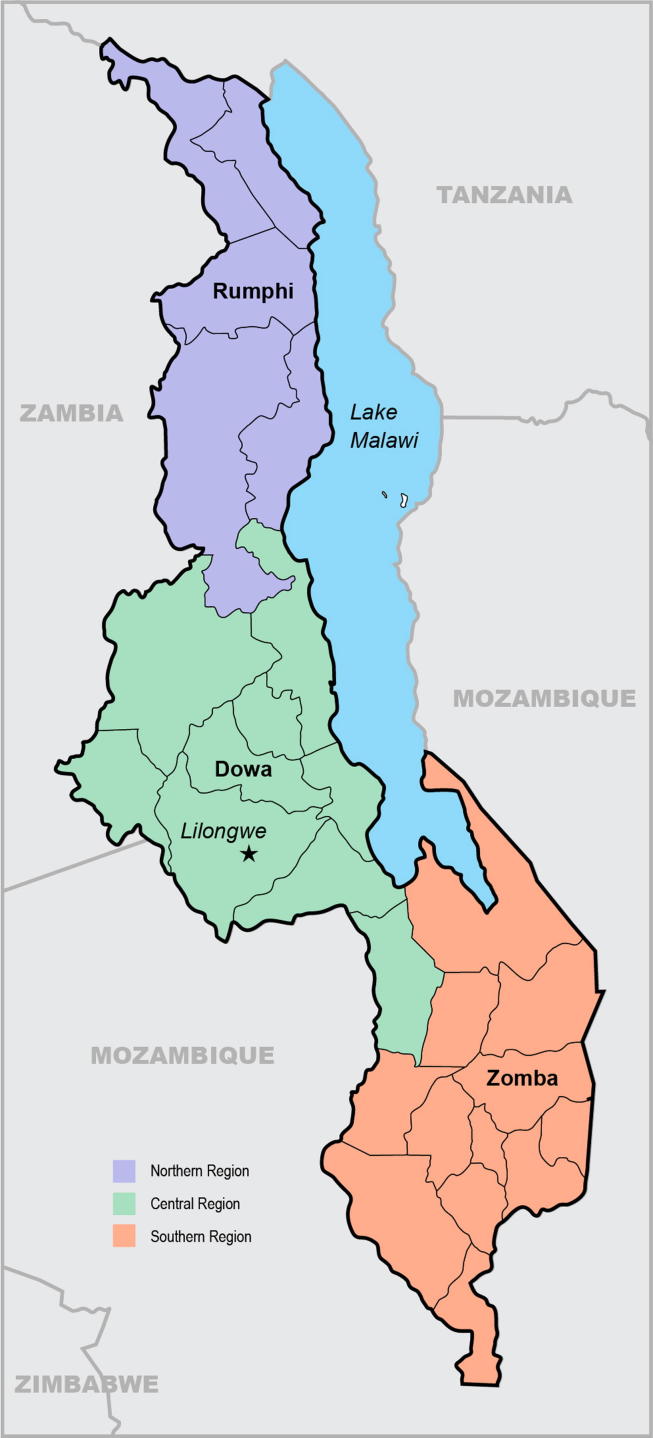
Map of study districts.

We conducted this study in one district in each of the three regions of Malawi: Rumphi (Northern Region), Dowa (Central Region), and Zomba (Southern Region) in 2015 (Fig. 1). We selected two health facilities and their surrounding catchment areas in each district to represent a mix of rural, peri-urban, and urban populations (Table 1).

**Table 1:** Background characteristics of study districts based on Malawi demographic health survey data, 2015–16 [11].

Region	Central		Northern		Southern	
District	Dowa		Rumphi		Zomba	
Health facility	Chisepo	Mponela	Mzokoto	St Patricks	Chamba	Matawale
Population density	Rural	Peri-urban	Rural	Peri-urban	Rural	Urban
TT2 + 1coverage^1^	76%		70%		71%	
Antenatal care received from skilled birth attendant^2^	97%		96%		92%	
Reported problem accessing health care^3^	77%		53%		73%	
Place of delivery^4^						
Health facility	91%		92%		91%	
Public sector	79%		80%		78%	
Private sector	12%		12%		13%	
Home	8%		6%		7%	
Other	1%		2%		2%	

1 Among women age 15–49 years with a live birth in the 5 years before the survey, percentage receiving two or more tetanus toxoid injections during the pregnancy for the last live birth.

2 Among women age 15–49 years who had a live birth in the 5 years before the survey, percentage receiving antenatal care from a skilled provider for the most recent birth.

3 Percentage of women age 15–49 years who reported having at least one problem accessing health care for themselves when they are sick.

4 Percent distribution of live births in the 5 years before the survey by place of delivery.

### 2.2 Study design and instruments

This was a mixed-methods, cross-sectional, descriptive study. The study protocol and data collection materials were developed with experts in survey and qualitative study design. We systematically collected qualitative data through separate focus group discussions with pregnant women and women pregnant within the last year (hereinafter referred to as “pregnant women”); male family members; and women likely to influence a pregnant woman’s health care decisions (mothers, mothers-in law, and sisters) (hereinafter referred to as “female family members”) (Table 2). We collected quantitative data from pregnant women leaving ANC clinics. We also interviewed non-users of ANC services. We conducted individual, semi-structured interviews with pregnant women, community leaders, lay community health workers, health workers providing care to pregnant women in health facilities, non-governmental development partners, district and national public health managers, and national policy makers. Table 2 describes key thematic areas covered with each group.

**Table 2:** Respondent groups, data collection methods, and key thematic areas explored.

Group	Data collection method	Number of groups or participants	Key themes
*Community members*			
Pregnant women	Focus group discussions	6	Knowledge of tetanus and influenza and their prevention Use of antenatal care services Acceptance of vaccination during pregnancy
	Individual, semi-structured interviews	19	Knowledge of tetanus and influenza and their prevention Use of antenatal care services Acceptance of vaccination during pregnancy Support for vaccination during pregnancy
	Individual exit interviews	55	Reason for health facility visit Support for vaccination and receiving health services during pregnancy Acceptance of tetanus toxoid vaccine Perception of health services received
Non-users of immunization services	Individual, semi-structured interviews	3	Knowledge of vaccines Reasons for not receiving vaccines Access to health care
Male family members of pregnant women	Focus group discussions	6	Knowledge of influenza, perceptions of risk and prevention Health care decision-making Barriers to health care for pregnant women
Females likely to influence pregnant woman’s health care decisions	Focus group discussion	6	Knowledge of influenza, perceptions of risk and prevention Health care decision-making Barriers to health care for pregnant women
Community leaders	Individual, semi-structured interviews	12	Knowledge of influenza, perceptions of risk and prevention Health care decision-making Barriers to health care for pregnant women
*Health professionals and policy-makers*			
Maternal child health staff at facility and community levels	Individual, semi-structured interviews	21	Priority of influenza disease Availability of health services for pregnant women Challenges to vaccine integration with antenatal care
Maternal child health managers at district and national levels	Individual, semi-structured interviews	4	Priority of influenza disease and availability of vaccine ANC as platform for vaccination Planning for new vaccine introduction
Expanded Program on Immunization (EPI) managers at district and national levels	Individual, semi-structured interviews	3	Target populations and integration between MCH and EPI Vaccine logistics and storage requirements Lessons learned from recent vaccine introductions
Health information system managers at district and national levels	Individual, semi-structured interviews	4	Monitoring and reporting maternal vaccination
National public health policy makers and partners	Individual, semi-structured interviews	10	Role in policy development Role in policy implementation and monitoring Policy environment for maternal influenza vaccine

### 2.3 Ethical considerations

The study met international ethics requirements and was approved by the ethical review boards of the Malawi National Health Science Research Committee (Lilongwe, Malawi) and the PATH Research Ethics Committee (Seattle, WA, USA). We obtained written informed consent from all participants prior to interviews and group discussions.

### 2.4 Inclusion criteria and sampling

Lay community health workers and nurses providing services to pregnant women in study areas were included. Community leaders and health workers identified pregnant women and family members for participation. We identified pregnant women who did not use immunization services through pregnant women and health providers participating in the study. We consulted public health stakeholders to identify national policy makers and non-governmental development partners. Focus group sample sizes were set with the expectation of reaching saturation, where no new issues emerge from additional respondents [15]. Inclusion criteria for participants included an age of at least 18 years, willingness to have the conversation recorded, and willingness to provide informed consent.

### 2.5 Data management and analysis

We conducted semi-structured interviews with national public health managers and policy makers in English; we conducted all other interviews and group discussions in local languages (*Chichewa* or *Chitumbuka)*. We audio recorded and transcribed interviews and discussions, and translated those conducted in local languages into English. Researchers collectively developed an analysis codebook based on interview guide themes and themes identified through an initial review of transcripts. We based our final coding structure on descriptive and interpretive codes using NVivo (Version 10, QSR International. Doncaster, Victoria, Australia) for thematic analysis. The results presented include themes and responses commonly expressed by participants; individual sentiments are identified when applicable.

## 3 Results

### 3.1 Participants

The study included 274 participants; 10% were district or national level health professionals, managers, or policy makers, and the remainder were pregnant women, female family members, or community immunization professionals (Table 2). Focus group discussions included between six and 12 participants. Despite a target number of 12–18 interviews with women who did not use immunization services, we were only able to identify three such individuals in our study areas. There were no refusals for participation.

### 3.2 Community knowledge of disease transmission and prevention

We asked pregnant women and male and female family members in focus groups about their knowledge of vaccine preventable diseases, specifically tetanus and influenza. For tetanus, while participants across focus groups correctly described symptoms of the disease (i.e., a newborn with “clenching fists”), we found high variability in understanding of its cause and transmission, with some participants identifying unhygienic birth practices and others suggesting sexual transmission. In all focus groups, there was a general understanding that tetanus vaccines can protect both a mother and her infant from disease. A typical explanation from one pregnant woman in Dowa suggested that she would be interested in being vaccinated during pregnancy, “*because the protection I will receive will also go to my baby.*”

In discussions about influenza, we found high variability at the community level regarding knowledge of both the disease and its vaccine. There is no direct translation of the word “influenza” in either of the study areas’ primary local languages, however recognition of “flu” was widely expressed by focus group participants after the term was introduced by facilitators. Facilitators subsequently linked “flu” to influenza. While community members were initially unable to distinguish influenza from a common cold, most respondents recognized influenza symptoms once described and the concept of airborne transmission of disease was well understood. In subsequent questioning, “flu” was the fifth most frequently mentioned condition by community members when asked to identify the most important health issue affecting pregnant women and infants (malaria was identified most often for both groups). Pneumonia was the second most frequently mentioned for infants. It was widely understood across focus groups that pregnant women and young children were more susceptible to disease, and participants frequently attributed this to having “lower immunity.” Suggestions from focus group participants for preventing the spread of influenza included keeping the house and environment clean, avoiding coughing on others, hand washing, and keeping homes well ventilated by opening windows.

### 3.3 Community acceptance of maternal immunization

We asked pregnant women, male and female family members, community leaders, and health workers about their perceptions of the safety and importance of maternal immunization. Citing their experience and knowledge of tetanus and TTV, pregnant women in focus group discussions across study sites expressed an overall positive view of maternal immunization. When asked if they would be interested in receiving an influenza vaccine when they were pregnant, two women explained that they would accept the vaccine “*so that our immunity will increase and the disease should not attack us*” and because “*you want to protect the unborn baby*”, which were typical responses. Of pregnant women interviewed while leaving health facilities after ANC visits, 95% (38/40) reported “no concern” regarding the safety of receiving a vaccine during pregnancy. In contrast to vaccines, traditional medicines and medicines sold informally or through unregulated “hawkers”, as opposed to those purchased through a health facility, were widely discredited as being unsafe for pregnant women.

When asked to identify concerns around vaccines administered to pregnant women, male and female family members, health workers, and managers suggested that new vaccines could be misperceived as contraceptives or may cause an abortion. A health worker explained that Depo-Provera is the most common injectable contraceptive used in Malawi and it is distributed through community health workers, like TTV, leading to concern that these similarities could cause confusion among community members. Managers speculated that other community members, particularly a woman’s male partner, may question why an important vaccine was not available to a broader population. Male family members also expressed dissatisfaction with the lack of vaccines specifically offered to men.

Pregnant women in focus group discussions across study sites generally did not express concern about receiving two vaccines at the same time during pregnancy; however, several women noted the importance of receiving information about the vaccines before being immunized. Despite general acceptance, some pregnant women described experiencing an adverse event following prior immunizations (a sore arm, swollen hands) and questioned whether symptoms would be more severe if two vaccines were given at the same time. A preference for receiving a combination vaccine instead of multiple single vaccine injections was a recurrent theme expressed. While not a common concern across focus groups, several pregnant women questioned the need for multiple doses of the same vaccine, indicating that the five-dose regimen of TTV for women of childbearing age was excessive.

### 3.4 Health care decision-making and support for pregnant women

We asked pregnant women to identify whom they sought for advice about receiving vaccines during pregnancy. In individual interviews, 46% of pregnant women identified community health workers as their main source of information on whether to receive vaccines, followed by friends or neighbors (34%) and doctors or nurses (29%). Across focus groups, pregnant women and female family members generally expressed strong support and respect for community health workers as trusted health care advisors. During focus group discussions with pregnant women, community leaders were also identified as important advisors for health care, and radio and newspapers were cited as sources of health care information.

When we asked male and female family members about whether they needed to provide “permission” in order for a pregnant family member to received health care, including vaccination, we found that it was commonly expected that a pregnant woman would have a discussion with her husband or male partner, or inform him of her intent to seek care, rather than formally request permission. Similarly, female family members described that while they participate in making decisions about the health care a pregnant daughter or daughter-in-law receives, they do not have the right to prohibit her from receiving health services.

### 3.5 Facilitators and challenges to receiving ANC, including maternal vaccines

When asked about what would encourage pregnant women to attend ANC, pregnant women in focus groups across sites regularly cited receiving general guidance around their pregnancies and specific information on the health of their babies and themselves. Receiving supplements, such as iron, was also mentioned by several women. Women frequently suggested that more respectful treatment by health care providers would encourage them to seek health care during pregnancy. Support from village leaders and community health workers was also mentioned as encouragement to seeking care.

We also asked family members how health services, including immunization services for pregnant women, could be improved. Common responses across sites included more respectful health providers and receiving clear information on the relevant diseases and the purpose of the vaccines, including their positive and negative attributes. A mother of a pregnant woman from Zomba described the importance of receiving information before receiving a vaccine, recommending a similar approach to the counseling that parents received around human papillomavirus virus (HPV) vaccine for their school-aged daughters: “*[It] is important to counsel the people the way they do with cervical cancer. They first call the parents and give them counseling so that they should make a decision as to who wants to vaccinate her child. Maybe some adults who are pregnant will not take part. It’s their freedom to do that.*”

We asked pregnant women and family members about challenges pregnant women experience in receiving health care and vaccination during pregnancy. All focus groups cited similar issues, none of which were specific to maternal vaccines. Challenges included health staff shortages, vaccine stock-outs, anxiety around HIV testing (which is routinely conducted during ANC), lack of respect for confidentiality on the part of health workers, lack of transportation, and poor treatment by health staff. Although not commonly mentioned across focus group discussions, several pregnant women cited a fear of the pain associated with receiving injections, indicating this was a motivation to avoid immunization. Several health care workers also mentioned pain from injections as a reason their patients do not attend ANC visits.

Non-users of immunization services were specifically sought in this study, and three women were identified and interviewed. All three attributed their non-use to membership in a religious group that they reported does not allow members to receive medical care from health facilities. One woman in Dowa explained, *“… [Members of my church] don’t take medication. So for all my ten children, I have not gone to receive this vaccine (TTV). Neither the children nor I have received the vaccine.”*

### 3.6 Perceptions of programmatic and operational factors important to maternal immunization

We asked health workers to describe programmatic factors of Malawi’s maternal health system that could support the delivery of new maternal vaccines. Health workers stated that the ANC system was designed to encourage family support of women receiving health care during pregnancy, including vaccination. Health workers explained that their clinics incentivized partner involvement in ANC by allowing women escorted by their male partners to be seen preferentially as compared to unaccompanied women. Health care workers also explained that they require women to receive TTV before they are offered any other ANC services, describing this as a key factor in promoting high immunization coverage. A common narrative across focus groups of pregnant women and male and female family members described community efforts to increase coverage of health services for pregnant women, including local laws requiring tetanus vaccination, ANC attendance, and delivery in a health facility. Pregnant women also described local government penalties if they missed ANC visits, including financial fines.

Study participants anticipated a number of operational challenges to introducing new maternal vaccines in Malawi. In discussions about the feasibility of adding a new vaccine to current vaccination coverage tracking systems, health workers and managers explained that while vaccination coverage is currently documented at the level of the community and health facility, information is not shared between healthcare sites, making it difficult to track vaccination status if a woman visits multiple health facilities during her pregnancy. Managers felt the current health information system had the capability to be expanded to capture coverage of a new maternal vaccine, although both health workers and managers voiced concern about the additional work required to track and report coverage monthly. Another concern raised at both district and national levels was the cost of reprinting health cards and registers to include dedicated space for tracking a new vaccine. Since influenza virus circulation is not routinely monitored outside of a few research sites in Malawi, accurate projections of the influenza season are not available. Without information on the duration of influenza season, maternal health care providers and immunization managers predicted difficulties in accurately estimating the number of women pregnant during, or just before, the influenza season for vaccine forecasting.

When we asked whether a formal system for tracking adverse events following immunization (AEFIs) was in place in the country, we received varied responses between sub-national and national stakeholders. Health workers reported that AEFIs were not systematically monitored for any vaccine, and one maternal health district manager explained that *“We do not have a [formal] system in place to record any adverse events, but women are advised that if they react to TTV, they should inform the TTV service providers at their local health facility.”* A ministry of health manager reported that Malawi did have a system for monitoring adverse events, but regarded it as “not aggressive,” citing a total of four serious adverse events reported to the national level from the district level in the previous three years.

In discussing anticipated challenges specific to maternal influenza vaccine introduction, public health managers noted concerns about the relatively short shelf life of seasonal influenza vaccine and the additional support required for proper disposal of unused vaccines at the end of each influenza season. A national-level manager recommended requiring vaccine suppliers to collect and properly dispose of expired and/or unused vaccines, citing the lack of government resources to do this on a national scale.

### 3.7 Recommendations for country-level maternal immunization planning

When presented with the hypothetical scenario of introducing seasonal influenza vaccine to pregnant women, maternal health workers identified several factors that they perceived could affect successful introduction. Adequate training was noted by health workers, district managers, and national policy makers as a key component of successful vaccine introduction in general, tying proper education and social mobilization for community members to increased vaccine acceptance and minimized misperceptions of vaccines. Education about seasonal influenza disease and the specific vaccine introduced was noted as particularly important, since influenza is not currently perceived as a leading disease priority of health workers or community members. A recurrent theme expressed by health workers was concern that sensitizing and educating the community about a new vaccine could lead to an increased workload for them. One health worker felt the additional tasks would negatively affect the quality of maternal health services, citing a perceived negative performance of community health workers after the introduction of HPV vaccine and a subsequent decline in overall vaccination coverage. Managers had differing opinions on the best approach to introducing a new vaccine. Some preferred staging the introduction to allow for applying lessons learned as services are nationally scaled and others preferred a nationwide introduction to avoid potential community perceptions of preferential treatment or discrimination.

We asked immunization managers at the district and national levels to identify the most important lessons learned from recent vaccine introductions in Malawi, including pneumococcal vaccine (2011), rotavirus vaccine (2012), and HPV vaccine demonstration projects in Zomba and Rumphi districts (2014). Sufficient long-term funding to implement the immunization program was a common priority for respondents, and adequate support for training health workers and social mobilization was specifically mentioned.

We asked health policy makers at the national level to provide recommendations for future introductions of new vaccines targeting pregnant women. Respondents highlighted the importance of advocacy and communication, and stakeholders felt that for maternal vaccines against influenza, communication efforts should focus on advocating the benefit of the vaccine to both the pregnant woman and her infant, instead of prioritizing one beneficiary over the other. Policy makers recommended simple, direct communication messages, noting the difficulty of translating more complex, nuanced messages into local languages. Health workers also cited the importance of having clear information on the side effects of any new maternal vaccine and were particularly interested in the safety of concomitant administration of any new vaccine with TTV.

With respect to decision-making around new maternal vaccines, respondents cited knowledge of local disease burden as an important consideration for prioritizing vaccines. Although preferred, policy makers reported that country-specific vaccine efficacy, safety, and cost effectiveness data were not required for decision-making, provided quality evidence from nearby countries was available. Respondents noted that decision-makers in Malawi have relied on data generated from countries with similar geographies and environmental and socio-economic indicators to inform previous public health decisions.

## 4 Discussion

This formative research study describes perceptions of maternal immunization from a variety of stakeholders in three districts of Malawi. It also identifies programmatic factors perceived to be important to the introduction and uptake of new maternal vaccines in the country and includes recommendations for maternal immunization planning at the country level. The information generated through this study can be used to assist public health decision-making and inform future vaccine introduction planning in similar contexts.

We identified several factors that could support the successful expansion of maternal immunization in Malawi, the first being that there is high health worker acceptance and community trust in vaccines in the study districts, including vaccines administered in pregnancy. This is advantageous for potential future introduction of maternal vaccines, given the importance of community acceptance to vaccine uptake [16], [17]. Second, vaccination was perceived as an integral part of ANC services by all groups of study participants. Third, health workers currently providing ANC services to pregnant women are experienced vaccinators.

With regard to influenza disease specifically, however, we found limited awareness and low priority of its prevention by pregnant women and health workers, indicating a strong need to educate the community, including preferred health advisors, on the disease and to sensitize stakeholders to the importance of the vaccine and its reassuring safety profile [18]. This would help dispel misconceptions, reduce vaccine resistance based on misinformation, and could be an avenue to support women who fear pain from injections. Implementing incentives to engage men in ANC and to encourage women to attend ANC visits has been successful. Further engagement of social networks and family members may be a useful strategy for gaining support for ANC attendance and vaccination during pregnancy and increasing awareness [19]. Targeted community education and engagement have been found to be a useful strategy in multiple developing country settings for gaining support for vaccine introduction [20], [21], [22]. As highlighted by policy makers interviewed in this study, increasing awareness that influenza vaccine benefits both a woman and her infant may play a part in ensuring vaccine acceptance, as women may prioritize the health of their infants over the benefit to their own health. While influenza vaccines benefit both mother and infant, effectively communicating the risk and benefit of maternal immunization for vaccines that provide a benefit only to the child's health and not the mother's needs to be further explored [23]. In the Malawi context specifically, it could be advantageous to engage certain faith communities which respondents report do not allow members to access health services and vaccination. While this study included very few non-users of immunization services, hesitancy to receive vaccinations and other health services may be higher among certain social or ethnic groups and may need to be addressed with targeted advocacy efforts.

In addition to community education, generating buy-in of key decision makers in Malawi will be valuable to the introduction of new maternal vaccines, but may be challenging for influenza given its currently low perceived priority compared to other diseases such as malaria and HIV. Participants in this study called for the strengthening of disease surveillance efforts to inform understanding of country-specific disease epidemiology, including identifying high-risk populations. The need to add influenza surveillance to the Malawi health information system was commonly mentioned by district and national respondents and is a key first step to gathering data needed to inform priority setting and awareness building. While there is a lack of detailed influenza epidemiology data in Malawi, limited surveillance is being conducted on influenza-associated hospitalized illness, influenza incidence in different populations, including pregnant women, and potential risk factors (such as HIV infection) for hospitalized disease [24], [25], [26], [27]. Additional efforts to strengthen and broaden surveillance could provide information identified by policy makers as important to informing public health immunization policies.

Surveillance systems for AEFIs are key to monitoring vaccine safety and addressing safety concerns in a timely manner [28]. Data on current vaccines used in pregnant women, including tetanus, influenza, and pertussis, is collected in large safety databases [19]; however, these AEFI surveillance systems typically do not accommodate the unique needs of maternal immunization safety monitoring [29], [30], [31], particularly in low resource settings. To assess relatedness, information on vaccine exposure, obstetric and maternal morbidities, and outcomes of both pregnant women and their offspring over time, in a linked fashion, must be captured. Such background information is rarely available in LMICs and safety monitoring in these settings is further challenged by the general lack of pharmacovigilance training, capacity, structures, and resources [32]. When this study was conducted in 2015, Malawi did not have AEFI surveillance guidelines or a formal committee to review cases. Nor did the country have a database for data collection on AEFIs or systematic training on AEFI surveillance. However, subsequent years have brought progress in this area, with some components of these systems now in place and training for EPI managers and healthcare workers specifically on AEFI surveillance ongoing [33]. As new vaccines are developed specifically for use in pregnant women, safety monitoring will be critically important for vaccine decision-making, introduction, and confidence [25]. Since adverse events occur in pregnancy that are unrelated to vaccination, systematic monitoring of pregnancy outcomes will be needed before new maternal vaccines are introduced in LMICs to provide baseline data on adverse events and to allow for accurate causality assessments.

Although TTV is currently integrated into ANC services in Malawi, additional research is needed to identify ways to minimize the strain that adding a new vaccine presents to health workers and health systems—concerns not specific to maternal vaccines, but raised by health workers and managers in our study—and whether it could provide benefit to ANC, as new resources or practices are brought in. This will be especially important to consider for maternal vaccines provided seasonally, such as influenza vaccines, as they require a short-term surge in the number of vaccines delivered and health system workload. The WHO updated guidelines for ANC services in 2016 [34], recommending an increase from four visits to eight per pregnancy. As this change is implemented in LMICs, it may provide an opportunity to strengthen ANC and integrate new maternal vaccines with ANC services. It will be essential, however, to ensure that new maternal vaccines are not replacing other essential services.

Our study has limitations. Although participants came from a variety of geographic areas in the country, the results and perspectives may not be representative of Malawi as a whole, or of other LMICs. However, we identified some similar themes in our study in a disparate population in Central America, particularly the high trust pregnant women place in community health workers and the importance of their advisory role in health care decision-making. Our sampling strategy, based on identifying pregnant women through community health workers providing maternal care and women leaving ANC clinics, captures only the experiences of women with access to health care. In addition, we were only able to identify three women in our study areas who refuse to use ANC services. Our results therefore may not be representative of women who chose not to access healthcare services or experienced significant barriers to healthcare access. However, we did attempt to identify respondents outside of ANC, a strength of our approach. Exploring experiences of populations with heightened challenges to healthcare access and potential strategies for outreach to them would provide additional valuable insight for vaccine introduction and delivery.

While Malawi’s experience achieving high maternal TTV coverage provides key lessons learned, additional information will be required to guide decision-making and priority setting around the introduction of new maternal vaccines, including influenza vaccine. Results of this study have been shared with Malawi’s Ministry of Health and can help inform both future vaccine introduction in Malawi and efforts to introduce new maternal vaccines or increase vaccination coverage in other LMICs.

## Declaration of Competing Interest

JAF, KMN, and NB received a sub-grant award from WHO to conduct this work; AM, BN, JK, MJP, and MS received support from PATH; and JO, PL and JH received grant funding from the Bill & Melinda Gates Foundation. KMN is a member of SAGE.

WHO disclaimer: Joachim Hombach and Philipp Lambach are staff members of the World Health Organization. The views expressed are those of the authors and not necessarily those of the World Health Organization.

## References

[1] Pertussis vaccines: WHO position paper August 2015. World Health Organization website. Available at http://www.who.int/wer/2015/wer9035.pdf?ua=1.u.

[2] Tetanus vaccines: WHO position paper February 2017. World Health Organization website. Available at http://apps.who.int/iris/bitstream/handle/10665/254582/WER9206.pdf;jsessionid=8F47BC3EFF97FFCB94946DBFF6C8C1A1?sequence=1.

[3] Vaccines against influenza: WHO position paper November 2012. World Health Organization website. Available at http://www.who.int/entity/wer/2012/wer8747.pdf?ua=1.

[4] Neuzil K.M. (2016). Progress toward a respiratory syncytial virus vaccine. Clin Vacc Immunol.

[5] Lin Shun Mei, Zhi Yong, Ahn Ki Bum, Lim Sangyong, Seo Ho Seong (2018). Status of group B streptococcal vaccine development. Clin Exp Vaccine Res.

[6] Ortiz J.R., Perut M., Dumolard L., Wijesinghe P.R., Jorgensen P., Ropero A.M. (2016). A global review of national influenza immunization policies: Analysis of the 2014 WHO/UNICEF Joint Reporting Form on immunization. Vaccine.

[7] World Health Organization (2018). Tetanus vaccines: WHO position paper, February 2017 - Recommendations. Vaccine.

[8] Bisset K.A., Paterson P. (2018). Strategies for increasing uptake of vaccination in pregnancy in high-income countries: a systematic review. Vaccine.

[9] Gkentzi D., Katsakiori P., Marangos M., Hsia Y., Amirthalingam G., Heath P.T. (2017). Maternal vaccination against pertussis: a systematic review of the recent literature. Arch Dis Child.

[10] Myers K.L. (2016). Predictors of maternal vaccination in the United States: an integrative review of the literature. Vaccine.

[11] Wong V.W., Lok K.Y., Tarrant M. (2016). Interventions to increase the uptake of seasonal influenza vaccination among pregnant women: a systematic review. Vaccine.

[12] World Health Organization. 2015. Meeting of the Strategic Advisory Group of Experts on immunization, April 2015: conclusions and recommendations. Weekly Epidemiologic Record, 90:261–280. Available at http://www.who.int/wer/2015/wer9022.pdf?ua=1. 26027016

[13] Fleming J.A., Baltrons R., Rowley E., Quintanilla I., Crespin E., Ropero A.M. (2018). Implementation of maternal influenza immunization in El Salvador: experiences and lessons learned from a mixed-methods study. Vaccine.

[14] Malawi demographic health survey 2015-16 https://dhsprogram.com/pubs/pdf/FR319/FR319.pdf; p. 121.

[15] Dworkin S. (2012). Sample size policy for qualitative studies using in-depth interviews. Arch Sex Behav.

[16] Dubé E., Gagnon D., MacDonald N.E. (2015). Strategies intended to address vaccine hesitancy: Review of published reviews. Vaccine.

[17] McArthur-Lloyd A., McKenzie A., Findley S.E., Green C., Adamu F. (2016). Community engagement, routine immunization, and the polio legacy in northern Nigeria. Glob Health Commun.

[18] Keller-Stanislawski B., Englund J.A., Kang G., Mangtani P., Neuzil K., Nohynek H. (2014). Safety of immunization during pregnancy: a review of the evidence of selected inactivated and live attenuated vaccines. Vaccine.

[19] Wilson R.J., Paterson P., Jarrett C., Larson H.J. (2015). Understanding factors influencing vaccination acceptance during pregnancy globally: A literature review. Vaccine.

[20] Baleta A.F., van den Heever J., Burnett R.J. (2012). Meeting the need for advocacy, social mobilization and communication in the introduction of three new vaccines in South Africa -successes and challenges. Vaccine.

[21] Schoub B.D. (2012). Introduction of inactivated polio vaccine (IPV) into the routine immunization schedule of South Africa. Vaccine.

[22] Vandelaer J. (2003). Tetanus in developing countries: an update on the Maternal and Neonatal Tetanus Elimination Initiative. Vaccine.

[23] Chamberlain A.T., Lavery J.V., White A., Omer S.B. (2017). Ethics of maternal vaccination. Science.

[24] Peterson I., Bar-Zeev N., Kennedy N., Ho A., Newberry L., SanJoaquin M.A. (2016). Respiratory virus–associated severe acute respiratory illness and viral clustering in Malawian children in a setting with a high prevalence of HIV infection, malaria, and malnutrition. J Infect Dis.

[25] Sambala E.Z. (2015). Vaccine effectiveness and coverage against the 2009 pandemic influenza in Ghana and Malawi. Disaster Med Publ Health Prepared.

[26] Ho A., Aston S., Jary H., Mitchell T., AlaertsM Menyere M., Mallewa J. (2016). Impact of HIV on the burden and severity of influenza illness in adults in Malawi: a cohort and case-control study. The Lancet.

[27] Divala T., Kalilani-Phiri L., Mawindo P., Nyirenda O., Kapito-Tembo A., Laufer M.K. (2016). Incidence and Seasonality of Influenza-Like Illnesses Among Pregnant Women in Blantyre, Malawi. Am J Trop Med Hyg.

[28] Bollyky T, Stegachis A. A report of the safety and surveillance working group; 2013. Retrieved from https://docs.gatesfoundation.org/documents/SSWG Final Report 11 19 13_designed.pdf.

[29] Cassidy Christine, MacDonald Noni E., Steenbeek Audrey, Top Karina A. (2015). Adverse event following immunization surveillance systems for pregnant women and their infants: a systematic review. Pharmacoepidemiol Drug Saf.

[30] Global Manual on Surveillance of Adverse Events Following Immunization. World Health Organization Website. Available at http://www.who.int/vaccine_safety/publications/Global_Manual_revised_12102015.pdf?ua=1.

[31] Causality assessment of an adverse event following immunization (AEFI): user manual for the revised WHO classification (Second edition). Geneva: World Health Organization; 2018. License: CC BY-NC-SA 3.0 IGO.

[32] Lackritz E, Stergachis A, Stepanchak M. 2017. Maternal immunization safety monitoring in low- and middle-income countries: a roadmap for program development. Seattle, WA. Retrieved from: http://gapps.org/docs/MaternalImmunizationSafetyMonitoringInLMICs.pdf.

[33] Personal communication, Olga Menang, PATH. April 2018.

[34] World Health Organization. 2016. WHO Recommendations on antenatal care for a positive pregnancy experience. Geneva, Switzerland: World Health Organization. Available at http://apps.who.int/iris/bitstream/handle/10665/250796/9789241549912-eng.pdf;jsessionid=5193F347028F97DFACE97B60199FCEE9?sequence=1. 28079998

